# Synthesis of Photoactive Materials by Sonication: Application in Photocatalysis and Solar Cells

**DOI:** 10.1007/s41061-016-0062-y

**Published:** 2016-08-10

**Authors:** Juan C. Colmenares, Ewelina Kuna, Paweł Lisowski

**Affiliations:** Institute of Physical Chemistry of the Polish Academy of Sciences (PAS), Kasprzaka 44/52, 01-224 Warsaw, Poland

**Keywords:** Photoactive materials by sonication, Photocatalysis, Ultrasounds, Solar cells, Perovskites, Quantum dots

## Abstract

In recent years, a good number of methods have become available for the preparation of an important group of photoactive materials for applications in photocatalysis and solar cells. Nevertheless, the benefits derived from preparing those materials through unconventional approaches are very attractive from the green chemistry point of view. This critical review work is focused on sonication as one of these promising new synthetic procedures that allow control over size, morphology, nanostructure and tuning of catalytic properties. Ultrasound-based procedures offer a facile, versatile synthetic tool for the preparation of light-activated materials often inaccessible through conventional methods.

## Sonochemical Preparation of Photoactive Catalytic Materials

The phenomenology of fabrication of highly efficient photocatalysts through an ultrasound route represents a very interesting and important area in science and technology, and it holds great potential for photocatalysts preparation in the near future [1–3]. In comparison with traditional sources of energy, ultrasound ensures unusual reaction conditions in liquid phase reactions due to the cavitation phenomenon (extremely high temperatures and pressures are formed in very short times in liquids) [3, 4]. Furthermore, application of ultrasound irradiation to a photocatalytic system might enhance both the bulk and localized mass transport and consequently expedite the molecular contact and the photocatalytic activity [1, 2, 4–6]. A considerable number of novel photocatalysts with novel nano-/microstructures (e.g., ZnWO_4_ [7], CdMoO_4_ [8], V_2_O_5_ [9], CuMoO_4_ [10], TiO_2_/Bi_2_O_3_ [11]) have been prepared by means of ultrasound-assisted methodology. In this subsection, we would like to focus the readers’ attention on ultrasound-promoted procedures for the preparation of photocatalysts, and the potential application of these materials in the photocatalytic degradation and selective oxidation of organic compounds.

### Sonochemical Synthesis of TiO_2_ Photocatalyst

The current status of outstanding progress made by titanium dioxide (TiO_2_), as the most widely used oxide semiconductor, has attracted considerable interest owing to its various applications in solar-driven hydrogen production, photocatalytic decomposition of pollutants, solar cells and so forth [12–14]. It is well known that overall efficiency for the solar-driven photocatalysis is very limited, because of its wide bandgap in the UV region, which accounts for less than 5 % of the total solar irradiation [15]. Currently, hydrogenated TiO_2_ nanoparticles (e.g., blue [16], brown [17] and black [18]) have opened a new avenue to the long-wavelength optical absorption and greatly enhanced photoactivity of the catalysts. Hydrogenated TiO_2_ is intensively investigated due to its improvement in solar absorption, but there are major issues related to its structural, optical and electronic properties, and therefore an easily compatible method of preparation is much needed [19]. Recently, disorder-engineered TiO_2_ nanocrystals treated in a hydrogen atmosphere under 20 bar of H_2_ atmospheres and 200 °C for approximately 5 days showed a colour change to black with a reduction in the bandgap energy up to ~1.54 eV [20]. It should be pointed out that band gap narrowing may be traced back to the surface disorder without the formation of Ti^3+^ centres. In other words, formation of surface disorders, oxygen vacancies, Ti^3+^ ions, Ti–OH and Ti–H groups, and band edge shifting are responsible for the optical properties and photocatalytic activity of black or hydrogenated TiO_2_. Interestingly, Ti^3+^ is not responsible for optical absorption of black TiO_2_, while there is evidence of mid-gap states above the valence band edge due to hydrogenated disorders [21–23]. It should be also noted that hydrogen doping induced a high density of delocalized Ti3d electrons leading to improved charge transport properties [24]. Furthermore, the hydrogenated black TiO_2_ exhibited highly efficient activity for the photocatalytic splitting of water [25]. Moreover, hydrogenation procedures need a high annealing temperature (over 400 °C) or high-pressure (e.g., 20 bar) and H_2_ is flammable and explosive [16, 17, 23–26]. The first attempt to prepare amorphous hydroxylated TiO_2_ with various degrees of blackness was based on the ultrasonic irradiation of high power intensity with enhanced photocatalytic activity of acid fuchsin degradation [27] (Table 1, Entry 1). A key feature of this system is that power density of employed ultrasonic irradiation was as high as 15 W mL^−1^, and a low ultrasonic power density could not make such changes in colour. Furthermore, ultrasound was employed to modify the original TiO_2_, which prepared amorphous hydroxylated TiO_2_ with black appearance, large surface area (328.55 m^2^ g^−1^) and enhanced photocatalytic methylene blue decomposition. It was found out that ultrasonic irradiation could accelerate the hydrolysis of TiO_2_ and reduce its particles size and form amorphous hydroxylated TiO_2_ by long time ultrasonication, giving rise a material with higher absorbance intensity through the whole visible light and near-infrared regions.

**Table 1:** Selected research studies of ultrasound assisted processes in the synthesis of photocatalysts and photoactive catalytic materials

Entry	Photocatalyst	Ultrasound source	Reaction conditions	Remarks	Refs.
1	Black TiO_2_	15 W mL^−1^	0.5, 1, 2, 4 and 8 h at 80 °C, 100 mL	Totally disorder structure of amorphous TiO_2_ both before and after ultrasonic treatment was observed	[27]
2	CaTiO_3_, BaTiO_3_, SrTiO_3_	45 kHz, 60 W	Ambient conditions for 10 h, glass tube sealed with a screw cap	CaTiO_3_ and BaTiO_3_ nanoparticles with almost regular spherical shape and uniform particle size (~20 nm) were observed. SrTiO_3_ particles were found to agglomerate more strongly leading to cubic-like aggregates with edge lengths varying (100–300 nm)	[31]
3	ZnO/Ag_3_VO_4_	12 mm diameter Ti horn, 75 W, 20 kHz	1, 1.5, 2, 3, and 4 h at RT, 150 mL	The nanocomposites prepared by 2 h ultrasonic irradiation have the best activity	[40]
4	ZnO/AgI/ Fe_3_O_4_	12 mm diameter Ti horn, 75 W, 20 kHz	0.5, 1, 2, and 4 h at RT, 150 mL	Photocatalyst prepared by ultrasonic irradiation for 1 hour has superior activity compared to other samples, and has remarkable stability and excellent magnetic filtration from the treated solutions	[41]
5	ZnO/AgI/ Ag_2_CrO_4_	12 mm diameter Ti horn, 75 W, 20 kHz	0.25, 0.5, 1, 2, and 3 h at RT, 150 mL	Material prepared by ultrasonic irradiation for 1 h has the superior activity	[42]
6	g-C_3_N_4_	12 mm, 33 Hz, 150 W	5 h at ambient temperature, 150 mL	g-C_3_N_4_ sheet possesses porous structure with high surface area and large pore volume	[43]

Worth mentioning is the work by Mao et al. [28] on the preparation of mesoporous titanium dioxide (TiO_2_) inside the periodic macropores of diatom frustules by sonochemical condensation of titania precursor, and then thermal treated at elevated temperatures, resulting in hierarchical macro/mesoporous materials. Furthermore, hierarchical structure provides a large number of accessible active sites for efficient transportation of guest species to framework binding sites. It was found that a high photocatalytic degradation of methylene blue as compared with P25 can be achieved with the composite having a certain loading amount of TiO_2_ (30 wt%), attributing to its hierarchical macro/mesoporous structures. It should be also noted that mesoporous titanium TiO_2_ with wormhole channel together with interconnected 3D structures inside diatom pores prepared by sonochemical synthesis with organic surfactant as structure directing agent has potentially important structural features for photocatalytic reactivity, because channel branching within the framework may facilitate access to reactive sites on the framework wall, which is most critical for its use as photocatalyst.

In terms of improvement of TiO_2_ photocatalytic properties under solar light by means of a suitable modification, enhanced photocatalytic activity of TiO_2_ nanocomposites for the degradation of dyes and Gram negative bacteria (*Escherichia coli*) was obtained by doped TiO_2_ with Al_2_O_3_, Bi_2_O_3_, CuO and ZrO_2_ [29] and synthesized via sonochemical method. It is interesting to notice that Bi_2_O_3_–TiO_2_ nanocomposite is more efficient toward the degradation of the dyes under solar light and TiO_2_ is the least efficient photocatalyst; the efficiency is in the order: Bi_2_O_3_–TiO_2_ > Al_2_O_3_–TiO_2_ > CuO–TiO_2_ > ZrO_2_–TiO_2_ > TiO_2_. It should be also noted that the antibacterial activity of all the five synthesized nanocomposites was tested against Gram negative bacteria (*E. coli*) by varying the concentrations (250, 500, 750 and 1000 µg). Among them, Al_2_O_3_–TiO_2_ shows some significant zone of inhibition around the film under dark condition. Recently, certain novel promising photocatalysts prepared by ultrasound-assisted method have been described in several research papers and review articles [1–3].

### Sonochemical Synthesis of Selected Photocatalysts

Sonochemical method has been used extensively to generate novel materials with unusual (photo)-catalytic properties, due to its unique reaction effects. For example, addition of titanium iso-propoxide induces the generation of new linear polymeric chains type nickel-titanium-ethylene glycol (Ni–Ti–EG), as revealed by the color change (from green to light blue). This light blue polymer coagulates to form a uniform rod-like precursor by van der Waals interactions [30]. Another elegant strategy to prepare ternary CaTiO_3_, SrTiO_3_ and BaTiO_3_ materials by a one-step room-temperature ultrasound synthesis in ionic liquid for photocatalytic applications (photocatalytic hydrogen evolution and methylene blue degradation) was recently described [31] (Table 1, Entry 2). It is also important to note that the use of ionic liquids in sonochemistry is advantageous due to their low measurable vapor pressure, low viscosity, low thermal conductivity, and high chemical stability, which are all significant parameters to generate efficient cavitation.

Photoactive materials such as SrZrO_3_have been recently explored because they exhibit suitable properties for photocatalytic hydrogen evolution from water splitting [32]. The results confirm that SrZrO_3_ was stable with time of hydrogen evolution and a very high rate (35 µmol g^−1^ h^−1^) was exhibited by SrZrO_3_ prepared by ultrasound-assisted synthesis. Recently, multiferroics such as BiFeO_3_ (BFO) are worthy of notice due to their unique and strong coupling of electric, magnetic, and structural order parameters in terms of practical applications, since both ferroelectric and antiferromagnetic ordering temperatures are well above room temperature (Curie temperature 830 °C and Neel temperature 370 °C) [33]. Moreover, other researchers have applied BFO materials prepared by sono-synthesis with high photocatalytic activity under solar light in degradation of various compounds such as Rhodamine B [34], methylene blue [35], Reactive Black 5 (RB5) [36] and phenolic compounds [37]. As anticipated above, BiFeO_3_ nanoparticles with measured band gap of 2.0 eV synthesized with ultrasound exhibited higher crystallization, smaller crystallite size and higher photocatalytic activity than the material synthesized with conventional methods.

**Fig. 1:**
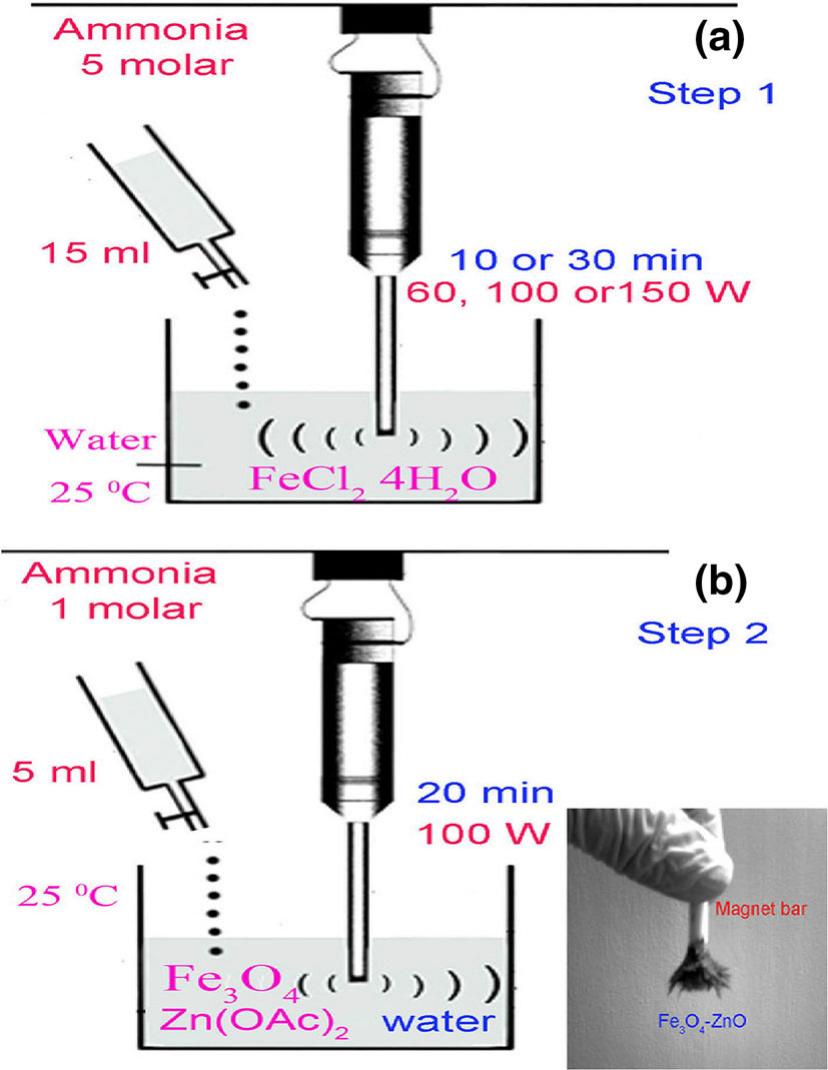
**a** Fe_3_O_4_ nanoparticles, **b** Fe_3_O_4_–ZnO nanocomposite prepared by surfactant-free sonochemical method. Reproduced from Ref. [38] with kind permission from Springer Science and Business Media

In order to improve the photocatalytic activity of a semiconductor and exploit its advantageous properties, Fe_3_O_4_/ZnO magnetic nanocomposites were synthesized via a surfactant-free sonochemical method in an aqueous solution (Fig. 1) and applied in the photo-catalytic degradation of various organic and azo dyes aqueous solution under UV light irradiation [38]. Surprisingly, these results showed that OH radicals generated by ultrasonic treatment were responsible for inducing the oxidation of Fe^2+^ into Fe^3+^. As soon as the required hydroxyl groups were available in the solution, the already-produced Fe^3+^ became saturated, and increasing the ultrasonic time did not help the dispersion of the formed nuclei. Additionally, prolongation of the reaction up to 30 min caused oxidation of most of the Fe^2+^–Fe^3+^ and as a result, brown FeOOH nanoparticles which are more likely to agglomerate were also obtained. Moreover, it was shown that ultrasonic power (100 W) had a significant but rather unpredictable influence on the mean magnetite particle size (agglomeration size decreased to 100–500 nm). Nanocomposites with 10 % of ZnO exhibited a super-paramagnetic behavior.

Previous research [39] showed that orthorhombic stannous sulfide (SnS) nanoparticles synthesized via a sonochemical route by 20 kHz sonication had smaller crystalline size (4 ± 1 nm) in comparison to the nanoparticles that were synthesized by 50 kHz sonication (6 ± 1 nm). An optical investigation confirmed that the SnS particles had strong emission bands located at the UV and visible regions, suggesting that they have the potential to be used as optical devices.

It is known that n–n heterojunctions between two n-type semiconductors can effectively promote separation of photogenerated electron–hole pairs, due to formation of internal electric field. Hence, preparation of ZnO/Ag_3_VO_4_ nanocomposites prepared by an ultrasonic-assisted one-pot method led to the enhanced photocatalytic activity for organic pollutants degradation under visible-light irradiation [40] (Table 1, Entry 3). Interestingly, photocatalytic activity of ZnO/Ag_3_VO_4_ nanocomposite under visible-light irradiation is about 21, 56, and 2.8-fold higher than that of the ZnO sample in degradation of rhodamine B, methylene blue, and methyl orange, respectively. Furthermore, it was revealed that the photocatalytic activity was attributed to greater generation of electron–hole pairs due to photosensitizing role of Ag_3_VO_4_ under visible-light irradiation and the efficient separation of the photogenerated electron–hole pairs due to formation of n–n heterojunction between the counterparts. Previous studies [41] (Table 1, Entry 4) showed that photocatalytic activity of the ZnO/AgI/Fe_3_O_4_ nanocomposite in degradation of rhodamine B, methylene blue, and methyl orange is about 32-fold higher than that of the ZnO/Fe_3_O_4_ sample prepared by ultrasonic irradiation method. The highly enhanced activity of novel ternary ZnO/AgI/Fe_3_O_4_ magnetic photocatalyst was mainly attributed to visible-light harvesting efficiency and decreasing recombination of the charge carriers as well. It should be noted that ultrasonic irradiation time and calcination temperature largely affect the photocatalytic activity. In another investigation [42] (Table 1, Entry 5), it was found that ternary ZnO/AgI/Ag_2_CrO_4_ photocatalyst with 20 % of Ag_2_CrO_4_ is active in rhodamine B degradation, with nearly 167-, 6.5-, and 45-fold higher conversion than that of ZnO, ZnO/AgI, and ZnO/Ag_2_CrO_4_, respectively, materials also prepared by ultrasonic irradiation method. Furthermore, visible-light activity of ZnO/AgI/Ag_2_CrO_4_ is about 6.5-, 16-, and 33-fold higher than that of the ZnO/AgI, whereas 45-, 22-, and 136-fold higher than that of the ZnO/Ag_2_CrO_4_ in degradation of rhodamine B, methylene blue, and methyl orange, respectively. It should be pointed out that photocatalyst prepared by ultrasonic irradiation for 60 min has the superior activity.

Recently, metal-free graphitic carbon nitride (g-C_3_N_4_) was synthesized via facile template-free sonochemical route and enhanced photodegradation of rhodamine B [43] (Table 1, Entry 6). The authors found that mesoporous g-C_3_N_4_ (112.4 m^2^ g^−1^) has almost 5.5 times higher photoactivity than that of bulk g-C_3_N_4_ (8.4 m^2^ g^−1^) under visible-light irradiation, which is attributed to the much higher specific surface area, efficient adsorption ability and the unique interfacial mesoporous structure that can favor the absorption of light and separation of photoinduced electron–hole pairs more effectively. Additionally, reactive oxidative species detection studies indicated that the photodegradation of rhodamine B over mesoporous g-C_3_N_4_ under visible-light is mainly via superoxide radicals.

Zhou et al. [44] prepared Zn_3_V_2_O_7_(OH)_2_(H_2_O)_2_ and g-C_3_N_4_/Zn_3_V_2_O_7_(OH)_2_(H_2_O)_2_ using sonochemical method with high photocatalytic activities in degradation of methylene blue. Interestingly, the absorption edge of g-C_3_N_4_/Zn_3_V_2_O_7_(OH)_2_(H_2_O)_2_ had red shift reaching 450 nm, and a significantly enhanced photocatalytic performance in degrading methylene blue, which increased to about 5.6 times that of pure Zn_3_V_2_O_7_(OH)_2_(H_2_O)_2_. During the ultrasound process, the heterojunction structure of g-C_3_N_4_/Zn_3_V_2_O_7_(OH)_2_(H_2_O)_2_ was formed and this special structure increased the separation efficiency of photogenerated electron–hole pairs, resulting in the enhancement of photocatalytic performances. Table 1 shows some detailed information about ultrasound assisted synthesis methods of photocatalysts made by selected research groups.

Another approach offering prospects for preparation of nanostructured TiO_2_/STARBON^®^-polysaccharide-derived mesoporous materials by means of ultrasound-assisted wet impregnation method was investigated [45]. Interestingly, carboxylic acid groups can be formed on the surface after initial thermal treatment at 400 °C under an oxygen-deficient atmosphere. Each carboxyl acid group acts as an individual nucleation site for TiO_2_ formation, which is possible under hydrolysis and condensation reactions promoted by sonication. Finally, the hybrid material (TiO_2_/STARBON^®^) is consolidated after thermal treatment at 400 °C. These conditions preserve pure, highly crystalline anatase phase (ca. 30 nm), leading to a reduction in the electron–hole recombination rate at the Starbon surface. TiO_2_ nanoparticles are strongly anchored and have good contact with the STARBON-800 structure (i.e., no leaching after 240 min of photocatalytic degradation of phenol), believed to enhance the photoelectron conversion (i.e., as compared with Norit and Graphene oxide supports) of TiO_2_ by reducing the recombination of photo-generated electron–hole pairs.

A similar study using a simple and effective ultrasound-assisted wet impregnation method was developed for the preparation of magnetically separable TiO_2_/maghemite-silica photo-active nanocomposites tested in the liquid phase selective oxidation of benzyl alcohol [46]. Interestingly, photocatalytic selectivity in organic media (90 % in acetonitrile) towards benzaldehyde was achieved at a benzyl alcohol conversion of ca. 50 %. Solvents played a significant role in the photo-oxidation process with materials showing very good conversion and selectivity in acetonitrile but not in aqueous conditions. Additionally, spatially ordered heterojunction between TiO_2_ and γ-Fe_2_O_3_ and a potential co-catalytic incorporation of Fe^3+^ into the TiO_2_ structure might significantly increase the sensitization and decrease the band gap energy of TiO_2_, effectively improving the photocatalytic activity and selectivity of these photocatalysts.

In an attempt to better understand the sonophotodeposition method (SPD), the iron-containing TiO_2_/zeolite-Y photocatalyst for the selective oxidation of benzyl alcohol was investigated [47]. Generally, photocatalyst prepared by the sonophotodeposition method showed better results, in terms of alcohol conversion and yield of benzaldehyde, in comparison with the photocatalyst prepared by an ultrasound-assisted wet impregnation method. Furthermore, sonication probe and sun-imitating Xenon lamp were tested, and for the first time, a non-noble metal was deposited on the TiO_2_ surface by using visible light, but with the help of ultrasound. It is worth noting that sonophotodeposition method is an innovative, simultaneous combination of ultrasonication and ultraviolet irradiation, and is highly energy efficient. Reactions can be carried out at room temperature and atmospheric pressure, very short reaction times and without using strong chemical reduction agents. Additionally, a considerable number of novel mono and bimetallic photocatalysts (e.g., Pd/TiO_2_ [48], Pd-Au/TiO_2_ [49], Pd-Cu/TiO_2_ [50]) have been prepared by sonophotodeposition methodology for selective oxidation of volatile organic compounds (VOCs).

### Ultrasonic Spray Pyrolysis for the Preparation of Photocatalysts

Ultrasonic spray pyrolysis (USP) method is a low-cost, continuous operation, and environmentally benign process with short processing time, which has been used to synthesize functional materials [51]. A distinctive feature of the method is that it can produce porous microspheres of various compositions without template and with good phase purity, and the morphology can be easily controlled during the process to ensure homogeneous composition distribution in the spheres [52, 53]. In this approach of ultrasonic spray pyrolysis, Suslick et al. [54] synthesized BiVO_4_ powders with particles ranging from thin, hollow and porous shells to ball-in-ball–type structures. Interestingly, materials prepared by USP are more active for oxygen evolving photocatalysts under visible-light irradiation (*λ* > 400 nm) in AgNO_3_ solution than commercial BiVO_4_ and WO_3_ powders, likely due to differences in the particle morphology. It was found that the increase of photocatalytic activity is likely due to the short distances electron–hole pairs must move to reach the surface in order to perform the desired redox reactions. Recently, USP was adapted to fabricate hierarchical porous ZnWO_4_ microspheres and evaluated by the degradation of gaseous NO_*x*_ under simulated solar light irradiation [55]. It was found that synthesis temperature (650–750 °C) was a key factor influencing the microstructures of resulting ZnWO_4_ samples, eventually affecting their photocatalytic activity, and which could be explained by improved optical absorption capability, high specific surface area, and fast separation/diffusion rate of the photogenerated charge carriers. Additionally, electron spin resonance spectroscopy (ESR) method indicated that O_2_^·−^ and ·OH radicals function as the major reactive species for NO_*x*_ decomposition. Compared to solid spheres, hollow PbWO_4_ spheres, which combine the merits of hollow and porous structures, could present improved mass transfer and high surface areas [56]. Moreover, hollow structured PbWO_4_ spheres exhibited superior photocatalytic activity to solid spheres (NO removal rate is 35 ppb min^−1^), due to the differences in microstructure and morphology.

**Fig. 2:**
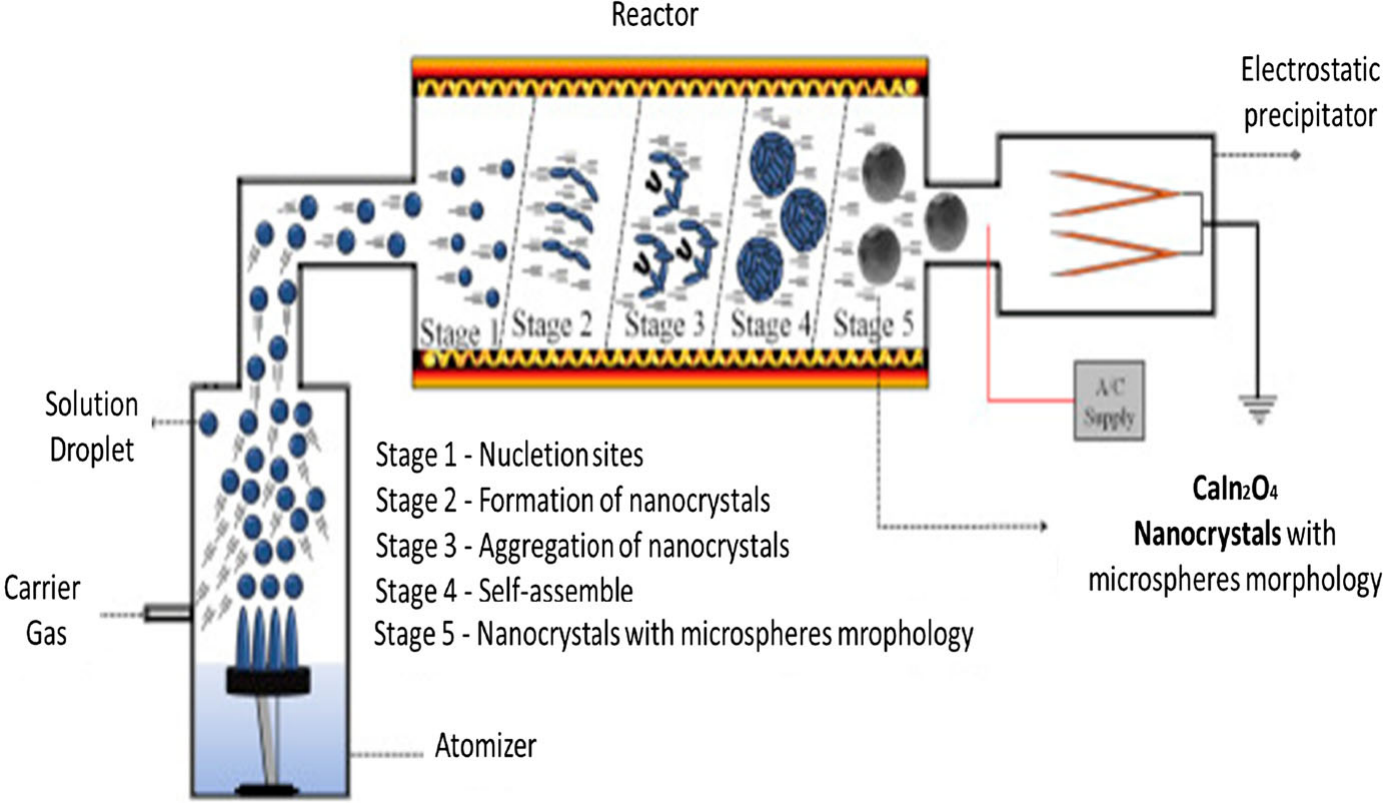
Schematic representation of CaIn_2_O_4_ nanocrystals prepared by ultrasonic spray pyrolysis. Reproduced and modified from Ref. [57] with kind permission from Springer Science and Business Media

Worth mentioning is the work by Tavares et al. [57] on the enhancement of the photocatalytic efficiency for the photodegradation of methylene blue applying the white emission of CaIn_2_O_4_ nanocrystals (band gap energy 3.83 eV) prepared by ultrasonic spray pyrolysis at 950 °C (Fig. 2). It should be noted that, USP provides a feasible approach for preparing shape- and size-controlled CaIn_2_O_4_ nanocrystals using short production times that hold great potential for photocatalytic applications and as photoluminescent materials capable of emitting white light. Moreover, the surface/bulk defects can influence the separation of photogenerated electrone–hole pairs on the CaIn_2_O_4_ under irradiation, and the purity of the products is high and the composition of the powders is easily controlled.

**Fig. 3:**
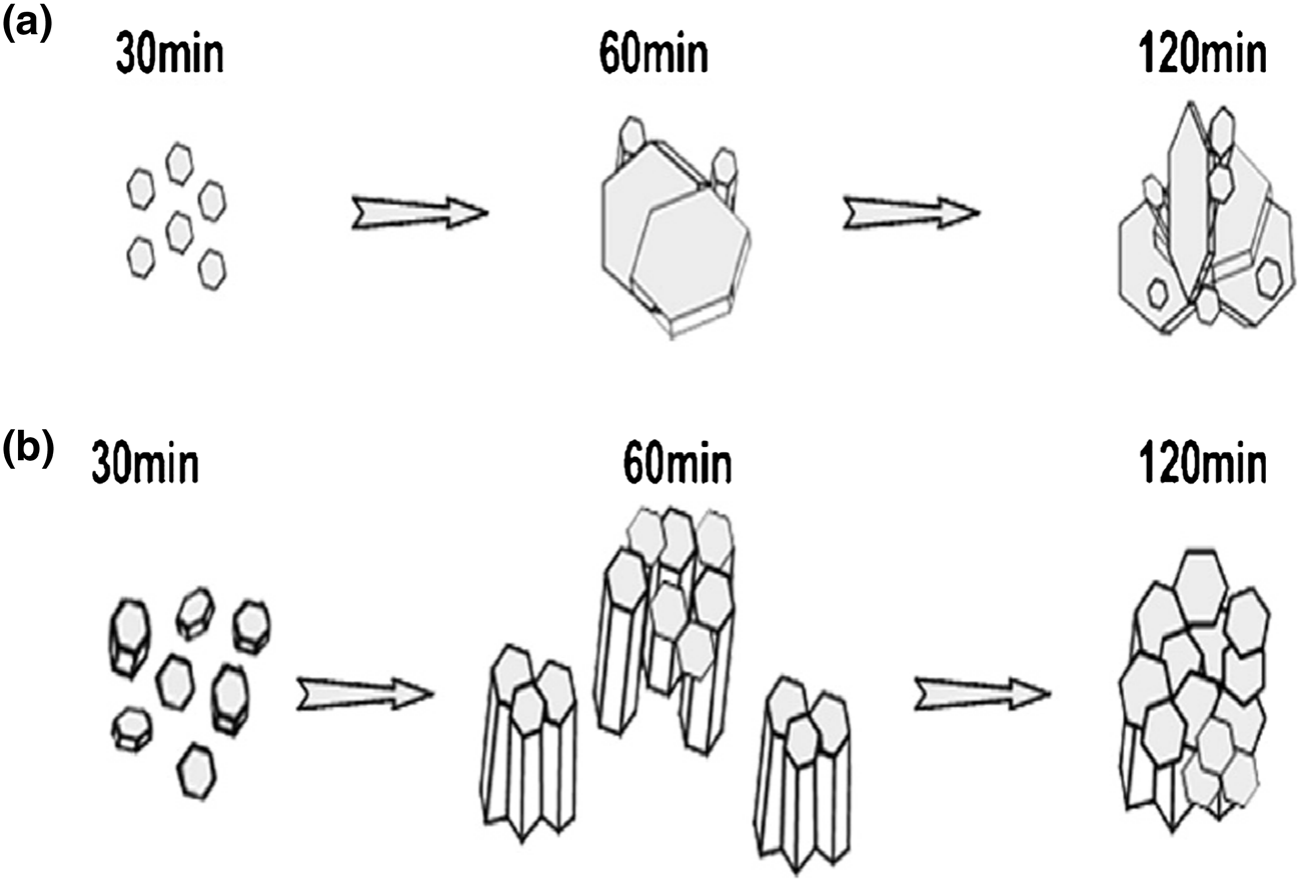
ZnO growth mechanism using ultrasonic spray pyrolysis after 30, 60 and 120 min of spraying time, using **a** 0.5 M Zn acetate, **b** 0.5 M Zn nitrate precursor solution. Reproduced from Ref. [58] with kind permission from Springer Science and Business Media

On the other hand, pure and Al-doped ZnO nanostructured thin films were grown at 400 °C on glass substrates by ultrasonic spray pyrolysis after 30, 60 and 120 min, using a 0.5 M zinc acetate precursor solution (Fig. 3a) and 0.5 M zinc nitrate precursor solution (Fig. 3b). The authors [58] found that both pure and Al-doped ZnO nanostructured thin films show good photocatalytic activity regarding the degradation of stearic acid under UV-A light illumination (365 nm), a behavior which is mainly attributed to their good crystallinity and the large effective surface area.

## Perovskite Solar Cells and Quantum Dots Photovoltaics

The interest in sonochemistry as a versatile tool for materials synthesis has been increasing, especially in the case of preparation of nanostructured materials for energy conversion like quantum dots, solar photovoltaic cells and dye-sensitized solar cells [59, 60]. The application of ultrasound allows to obtain a uniform shape and highly pure nanoparticles with a narrow size distribution, which has influence on the unique characteristics of photovoltaic devices. Additionally, ultrasound-assisted methodologies can decrease the time of synthesis, consequently reducing operating costs and develop effective manufacturing processes [60, 61]. Hence, it is a promising inexpensive alternative for the production of the next generation of solar cells such as quantum dot sensitized solar cells (QDSCs) or perovskite solar cells (PSCs) [62]. Perovskites are a class of compounds that possess well-defined crystal structure with formula ABX_3_, where, the A and B sites are engaged by cations and the X site is occupied by the anion. Depending on the type of the ions, they can form various perovskite crystal geometries with different features. The cations in the lattice are able to enhance and modify the band structure, which is particularly important from the point of view of photophysical properties [63, 64]. Thus, perovskites as an active layer in photovoltaic systems can deliver high open-circuit voltages and lead to the harvesting of light from a broad spectrum [64]. Perovskite solar cells are able to absorb the shorter wavelengths of visible light corresponding to photons with higher energy. Consequently, they can reach the maximum voltage of cells adequate to the maximum electrical power that comes from incident photons. Therefore, PSCs that stem from dye-sensitized solar cells (DSSCs) are considered as a forerunners of emerging photovoltaic technology [64, 65].

Perovskites first used for solar applications were documented in a seminal article by Miyasaka and co-workers in 2009 [66], and then by Park and co-workers 2 years later [67]. In all cases, the perovskite solar cells emerge as long-running, durable photovoltaic devices with high efficiency, and have been increasing from 10 to 20 % in the last few years [68, 69]. Due to fact that PCSs possess an enhanced light-harvesting system, improved charge separation process as well revised charge transfer and charge collection [68, 69], the power conversion efficiency (PCE) of perovskites solar cells is remarkable higher compared to the previous generation solar cells devices [i.e., wafer-based silicon devices or thin film SCs made from cadmium telluride (CdTe)]. The properties mentioned above can be attributed to the special structure of these materials, which mainly reflect on their application of photovoltaic technology as well as of other domains [70].

The next interesting possibility leading to more efficiently working solar cells is the development of quantum dot sensitized solar cells. It is worth noting that the energy conversion of solar cells directly depends on their structure, i.e., conductive substrates, semiconducting layers and in particular, sensitizing molecules (dye or quantum dots). The structure of DSSCs is similar to that of QDSCs; however, the power conversion efficiency (PCE) is remarkably lower in the case of QDSCs. Nevertheless, due to the relatively low cost related to using inexpensive semiconductors and a simple fabrication, possible approaches for improving PCE are still under debate [61, 62, 71].

In this subsection, we would like to focus the readers’ attention on the use of sonochemical methodology as a convenient tool for the preparation of solar cells with higher power conversion efficiency; indicating the influence of ultrasounds on the formation of quantum dots and perovskite solar cells, and pointing out the current state of the art.

### Sonochemical Synthesis of Perovskites and Quantum Dots

The wide range of sonochemical methods used for the synthesis of perovskite-type oxides ABO_3_ have been well reviewed by Colmenares group [72]. Among the most effective sonication-based processes, we can distinguish mainly co-precipitation methods, sol–gel methods, and hydrothermal methods as well aerosol synthetic techniques [e.g., the ultrasonic spray pyrolysis (USP)]. In comparison with conventional methodologies, which are mentioned above, the USP allows for control over size and shape of particles in a simple and more reproducible way. The selection of optimal parameters (such as ultrasound frequency, characteristic of the precursor solution and temperature) allows a nanomaterial with desirable properties to be obtained. However, it is not the only effective approach for the preparation of thin-films and ultrafine nanostructures in terms of manufacturing photovoltaic devices. An interesting concept that involves combination of ultrasonic spray technique and thermal evaporation process was reported by Xia et al. [73]. The modified deposition method assisted by ultrasonic spray coating used for the formation of CH_3_NH_3_PbI_3_ thin film layers enables control of morphology. The obtained perovskite film possesses larger crystal size (>500 nm), which result in higher carriers mobility and lower charge recombination. Thereby, uniform coverage prevents an undesirable short circuit between the top and back surface contacts of a solar cell, resulting in an increase in the power conversion of photovoltaic devices [73].

CH_3_NH_3_PbI_3_ nanoparticles (NPs) can also be synthesized by another sonochemical technique (Table 2; Entry 1) [74]. The method applied in this work involves ultrasonic irradiation for the preparation of ultrafine nanocrystal sensitizers for solar applications. The sonochemistry reaction is carried out in a non-aqueous solution, leading to the generation of nanoparticles with polygonal shapes in the narrow size-range. The sonochemical synthesis of methylammonium lead halide takes place only in isopropanol medium, and the presence of water reduces the reaction rate [74, 75]. Additionally, it has been shown that the morphology of the particles strictly depends on the irradiation time [74]. The nanoparticles obtained during 10 min of reaction exhibited irregular shapes, whereas the particles achieved after 20–30 min showed an opposite trend and formed hexagonal or triangles configuration [74]. Nevertheless, the shape of perovskites can be changed when the synthesis step is preceded by ultrasonic pretreatment of the precursor solution. Kesari and Athawale [75] demonstrated that ultrasound-assisted method arranged to CH_3_NH_3_PbI_3_ synthesis allows to obtain a rod shape and a tetragonal crystal structure. Results indicate that the preparation route has an important impact on a sample’s morphology and hence on its optical properties. It has been found that the band gap of the CH_3_NH_3_PbI_3_ hybrid materials synthesized by ultrasonic method (from standard 1.5 to 2.25 eV) shifted into the blue region, which can be a sign that the materials possess features of quantum dots [74]. A similar behaviour of NaTaO_3_ perovskite was observed by Vázquez-Cuchillo et al. [76], when the crystalline materials were more light sensitive in the UV-range compared to the same materials obtained by other technique (i.e., solid-state method). On the other hand, the defects forming on the surface due to cavitation effects can also turn to light electron capture centres and decrease the band-gap energy, which is rather more favorable from the photocatalytic point of view [77].

**Table 2:** Selected examples of sonochemical methods applied for the synthesis of active solar cells layers

Entry	Ultrasound source	Key parameters	Remarks	Refs.
**Perovskites**				
1	The ultrasonic transducer (frequency 20 kHz) was operated at an amplitude of 60 %	Time of irradiation increased from 10 to 30 min	The CH_3_NH_3_PbI_3_ nanoparticles of polygonal shapes in the size range of 10–40 nm were obtained until 20 min reaction, whereas the shorter time of irradiation resulted in non-uniform shapes	[74]
2	A multiwave ultrasonic generator equipped with titanium oscillator (12.5 mm) operating at 20 kHz	Power of ultrasound was adjusted in the range from 50 to 70 W	The particle size of CuInS_2_ was about 100 nm when the power of ultrasound was equal to 50 W—increasing the power led to formation of uniform particles with size of 60 nm	[81]
3	A sonication system operated at 59 kHz and a power output of 99 W	Concentration of precursor and sonication time (various variants)	The desired kesterite phase of CuZnSnS_2_ was obtain using longer time of irradiation and higher precursor concentration	[82]
**Quantum dots**				
4	A ultrasound probe (frequency 20 kHz, 130 W/cm^2^) operated at 50 % amplitude	The effect of ultrasound on the reduction process	The reduction process of tellurium does not exceed 15 min and allows to obtain CdTe QDs with the band gap value (2.3 eV)	[85]
5	Ultrasound horn with 1.9 cm diameter (20 kHz, output acoustic power 45.5 W)	The sonication time (0–45 min) and temperature (40–60 °C)	The CdS nanoparticles have uniform spherical morphology with size around 2 nm (60 °C after 45 min of irradiation).	[86]

**Fig. 4:**
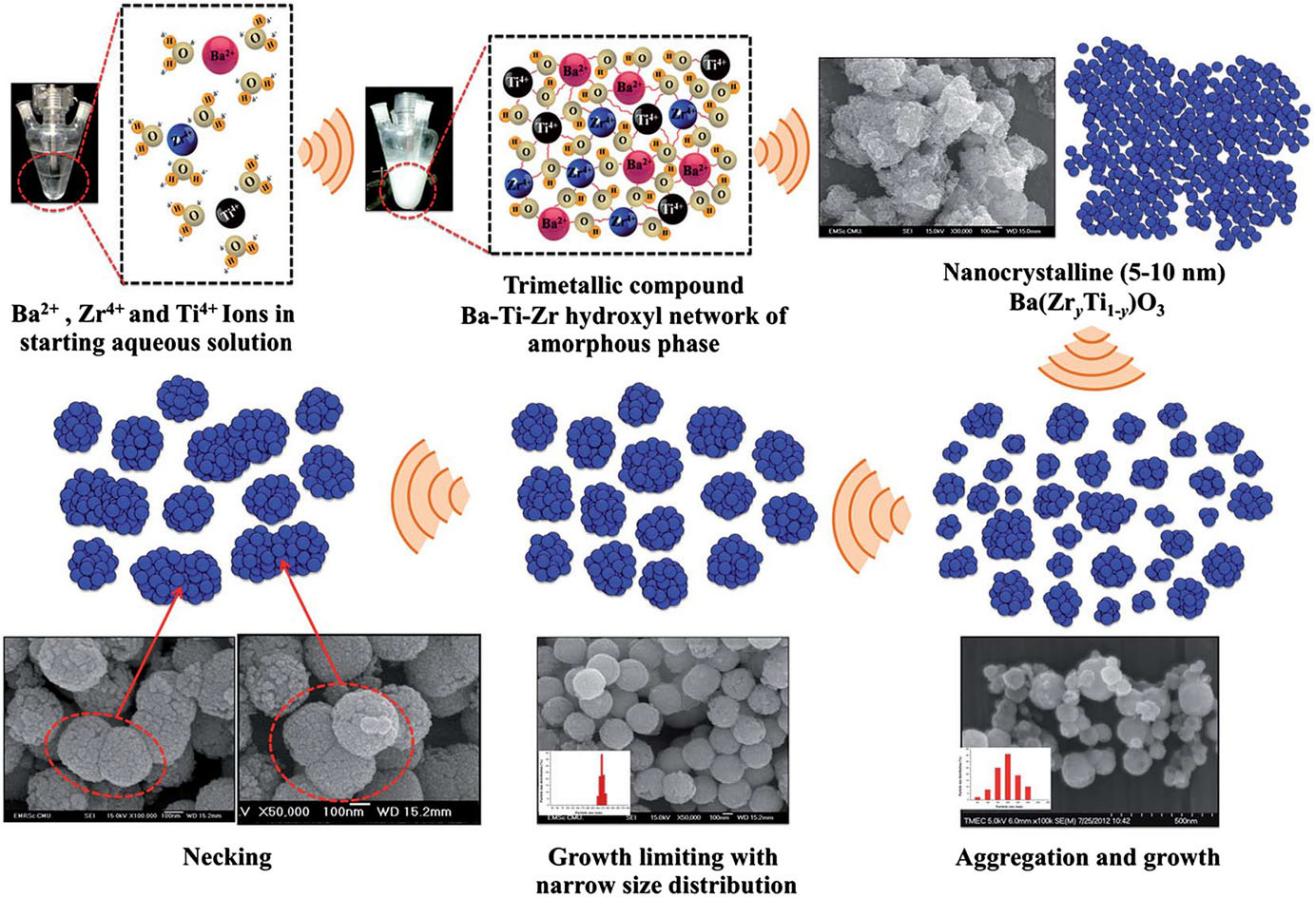
Schematic diagrams illustrating formation of the crystal growth mechanism. Reproduced from [80] with permission of The Royal Society of Chemistry

The application of ultrasound in perovskites’ synthesis allows to obtain the required products using low temperature synthesis methods. The great number of cavitation active sites generated under ultrasonic irradiation led to materials that possess a small particle size and a uniform bulk [77, 78]. However, the formation and growth of nanosheets and face-like particles depend on ultrasound as well as on calcination conditions. The calcination temperature affects the crystallinity, microstructure, optical and dielectric features of the samples. Consequently, the particles that were synthesized under the same ultrasonic conditions and calcined at different temperatures exhibited diverse properties as proved by various researcher groups [79, 80]. Interesting is the fact that contrary to previous studies, Wirunchit et al. [80] presented a facile sonochemical synthesis of perovskite oxides without any sintering and calcination step (Fig. 4). In this case, the formation of nanoparticles with a spherical morphology and a narrow particle size distribution occurs through the sonocrystallization process, whereas the effect of ultrasonic irradiation generates and promotes the nucleation as well as inhibits or delays the crystal growth process [80]. Furthermore, the crystal size and morphology of nanostructures also depend on the power energy of ultrasound (Table 2; Entry 2). Results presented by Amiri et al. indicate that increasing the power of ultrasound causes the production of uniform CuInS_2_ nanoparticles with small size (~60 nm), whereas using the lower ultrasonic energy led to the creation of bigger aggregated particles with a lump-like structure [81].

The one-step sonochemical method proposed by Liu et al. [82] can be applied for the production of Cu_2_ZnSnS_4_ perovskites (Table 2; Entry 3). By this way, the propitious shape of nanoparticles can be obtained by facile and rapid synthesis through the selection of an optimal precursor’s concentration as well the above-mentioned sonication time [82]. However, in the event of the production of high quality inks for solar-cells fabrication, a more suitable methodology seems to be the ultrasound-assisted microwave solvothermal method. Unlike the others, the nucleation–dissolution–recrystallization mechanism allows to obtain Cu_2_ZnSn_4_ nanosized particles with hexagonal prisms structure, due to fact that the synergistic effect of ultrasound and microwave plays a key role in the growth process of wurtzite phase [83, 84].

Ultrasound-assisted processes are also used to prepare quantum dot particles (QDs) for solar cells application. The simple and fast sonochemical methodology provides monodispersed CdTe QDs with a strong quantum confinement regime (Table 2; Entry 4). The application of ultrasound allows one to control the morphology and reduce the surface defects of quantum dots. As a result, this allowed the production of nanoparticles with only one fluorescence band, which is beneficial in terms of photovoltaic devices [85]. Furthermore, it is clearly shown that the application of ultrasound, in particular the sonication time, has an influence on the particle size distribution and the growth kinetics (Table 2; Entry 5) [86]. Results indicate that the increase of particle size at longer time is attributed to the diffusion-limited coarsening process, which is accelerated by cavitation. Thus, the effect of ultrasound irradiation causes that the synthesis of QDs nanoparticles occurs in shorter time using low temperature methodology such as the micro-emulsion method [86]. Additionally, sonochemically synthesized QDs possess better optical properties and are able to produce a large amount of reactive oxygen species (ROS), which may influence a further course of the photochemical processes [87].

### Sonochemical Solar Cells Fabrication

Sonochemical methodology can be used as the first step toward the synthesis of photoactive layers or as the second step for the preparation of solar cells devices. The wide range of ultrasound parameters (i.e., ultrasonic power, frequency and time) have a positive influence on the size, morphology, structure or the surface area, and in consequence on optical as well as electrical properties of photovoltaic devices [81]. It was proven that the ultrasound irradiation accelerates the nucleation process, and promotes ionic and mass diffusion, which permit the synthesis of structures with desirable features in terms of the photovoltaic application [88]. For this reason, ultrasounds play a crucial role in the preparation of solar cells structures and cells’ components.

The photoactive layers can consist of perovskite nanoparticles, quantum dots or hierarchical hollow spheres which can improve the efficiency of dye-sensitized solar cells (DSSCs), or quantum dots solar cells [89]. The DSSCs type of solar cells exhibited significant better parameters (i.e., a short circuit current density, open circuit voltage and fill factor) in comparison to standard nanoparticle photoelectrode, consequently increasing the power conversion efficiency and photocatalytic performance [90]. The hierarchical structures (HSs) (mainly ZnO, TiO_2_, SnO_2_) synthesized through the sonochemistry methodology demonstrate required properties caused by their nano/micro combined architectures. They enable fast electron transport due to an ideal network created by nanosheets connection. Additionally, HSs possess a large surface area, which improves the accessibility for incident photons [88, 91]. Furthermore, dye-sensitized solar cells based on hierarchical structures are able to enhance the light harvesting capability and improve the efficiency of SCs [91]. Using ultrasound treatment technique (UST), the change of the solar power efficiency can be improved up to 50 % [92].

**Fig. 5:**
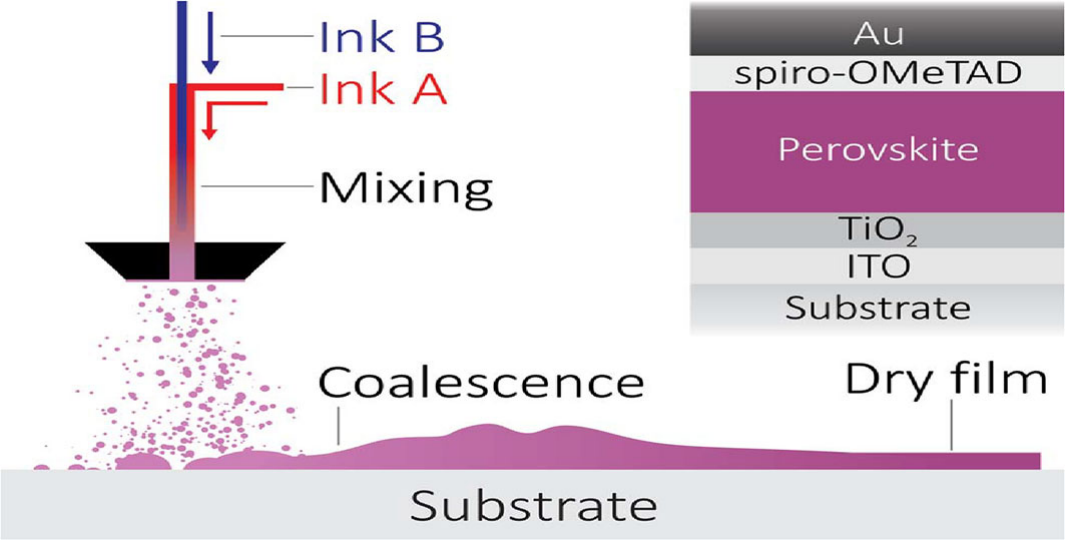
Schematic of pumped ultrasonic spray coating for perovskite precursor deposition. Reproduced from [94] with permission of The Royal Society of Chemistry

Among many sonochemical methods used for the fabrication of solar cells, we can spot the ultrasonic spray coating method (USP). The USP allows one to deposit uniform perovskite thin films on glass substrates (Fig. 5) [93, 94]. The formation of dense films having a surface coverage above 85 % guarantees maximum device efficiency. For this reason, the USP coating leads to an increase of the power conversion efficiencies of perovskite-based photovoltaics devices [94]. Concurrently, ultrasonic spray coating with various rates provides the quick optimization of thickness, precursor ratios and resulting pinhole-free layer crystallinity [95]. Furthermore, USP provides high-performance flexible perovskite solar cells by using a combination of ultrasonic spray-coating and low thermal budget photonic curing [94].

**Fig. 6:**
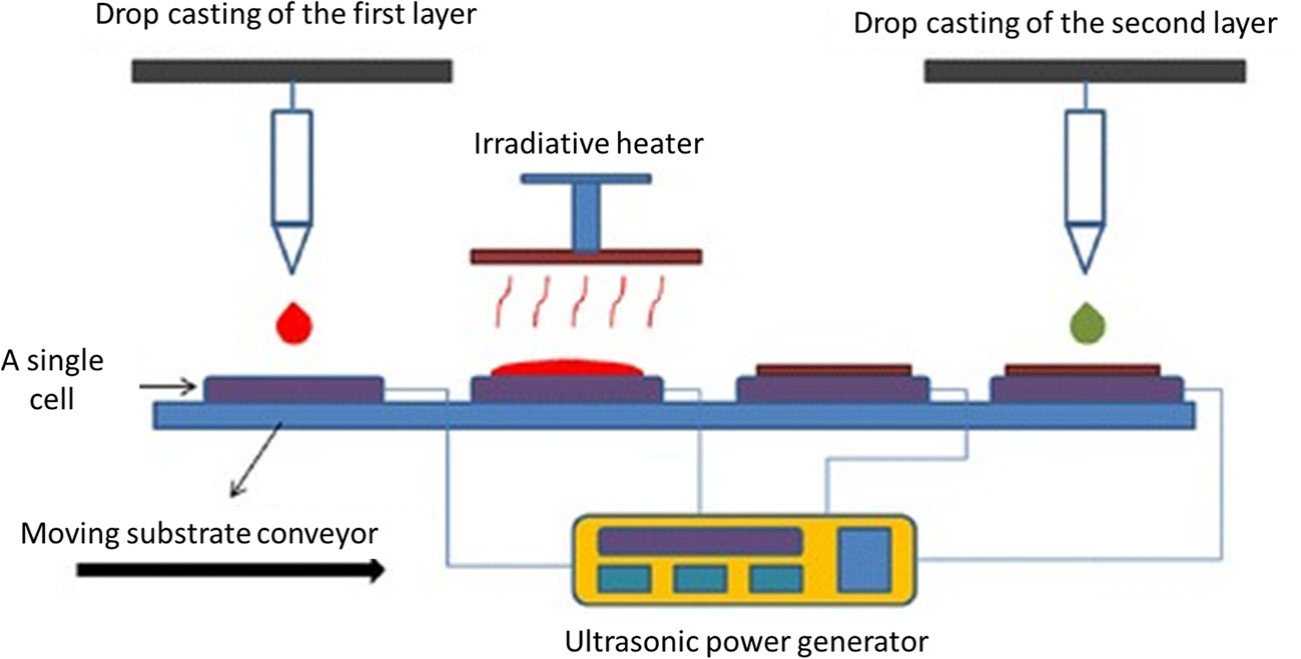
Scheme of the automated manufacturing of SVADC process for solar cells fabrication. Reproduced and modified from Ref. [96] with kind permission from Springer Science and Business Media

As the next promising tool for the fabrication of micro-thin as well as nano-thin films, the substrate vibration-assisted drop casting method (SVADC) has emerged. Due to fact that the SVADC is scalable casting methodology, it can be used to prepare an array of thin-film solar cells, perovskite, and quantum-dot solar cells or other thin-film devices through an automated fabrication process (Fig. 6). The same manufacturing process allows several layers of a thin-film solar cell to be deposited. It is worth to note that the solution properties, surface wettability, impingement conditions as well as substrate vibration have influence on the maximum effective and uniform surface of solar cells. Due to the fact that this method allows one to obtain a simple structure (possessing efficiency over 3 %) without requiring an optimization process and the application of expensive materials or treatments, the SVADC might replace the old generation techniques of ultrasound-assisted methods used for the fabrication of silicon wafers [96].

## Abbreviations

UV: Ultraviolet
BFO: Bismuth ferrite
SPD: Sonophotodeposition
RT: Room temperature
RB5: Reactive black 5
SnS: Stannous sulfide
VOCs: Volatile organic compounds
USP: Ultrasonic spray pyrolysis
ESR: Electron spin resonance spectroscopy
QDSCs: Quantum dot sensitized solar cells
PSCs: Perovskite solar cells
PCE: Power conversion efficiency
DSSCs: Dye sensitized solar cells
CdTe: Cadmium telluride
NPs: Nanoparticles
QDs: Quantum dots
ROS: Reactive oxygen species
HSs: Hierarchical structures
UST: Ultrasound treatment technique
SVADC: Substrate vibration-assisted drop casting method
ITO: Indium tin oxide
spiro-OMeTAD: *N*_2_,*N*_2_,*N*_2_^′^,*N*_2_^′^,*N*_7_,*N*_7_,*N*_7_^′^,*N*_7_^′^-octakis(4-methoxyphenyl)-9,9^′^-spirobi[9H-fluorene]-2,2^′^,7,7^′^-tetramine
